# Supplementary material for: A widely distributed family of eukaryotic and bacterial deubiquitinases related to herpesviral large tegument proteins
Source: Nat Commun. 2022 Dec 10;13:7643. doi: 10.1038/s41467-022-35244-y (PMC9741609; doi:10.1038/s41467-022-35244-y)

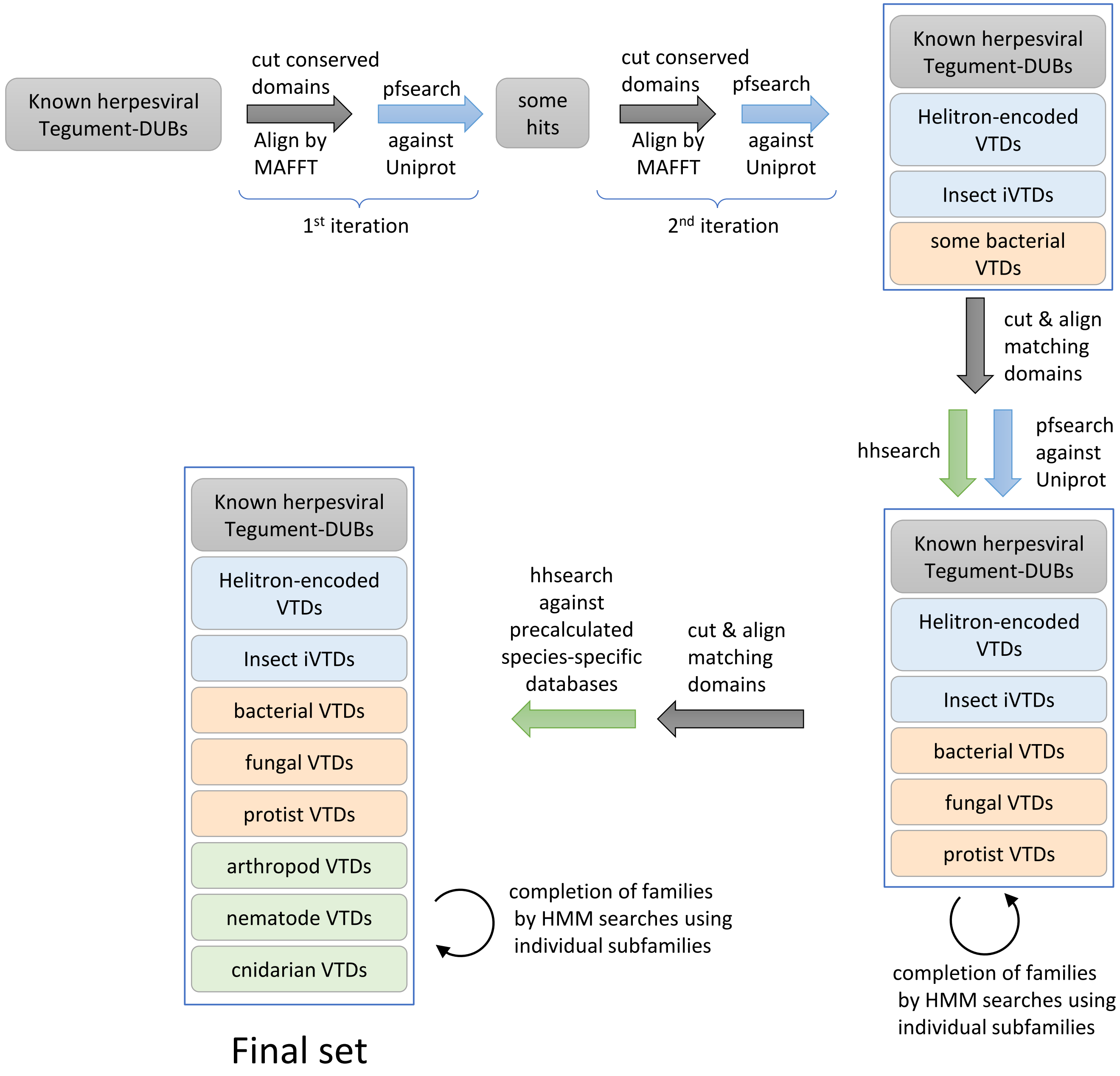


**Supplementary Fig 1**

Graphical representation of the search strategy. The methods are represented by arrows, the sequence sets identified are represented by colored boxes. Starting from viral tegument DUBs (grey box), two rounds of ‚pfsearch' searches find helitron-coded VTDs an insect iVTDs (blue boxes). A few bacterial VTDs were already found in this search, the rest – together with fungal and protist VTDs (orange boxes) are identified in a successive search using pfsearch and hhsearch. The final sequence set, including arthropod, nematode and cnidarian VTDs (green boxes) is obtained after additional hhsearch searches. Some of the more divergent members of the VTD family are not found by profiles/HMMs calculated from all available sequences. In these cases, subfamily-specific profiles and HMM help to complete these families.

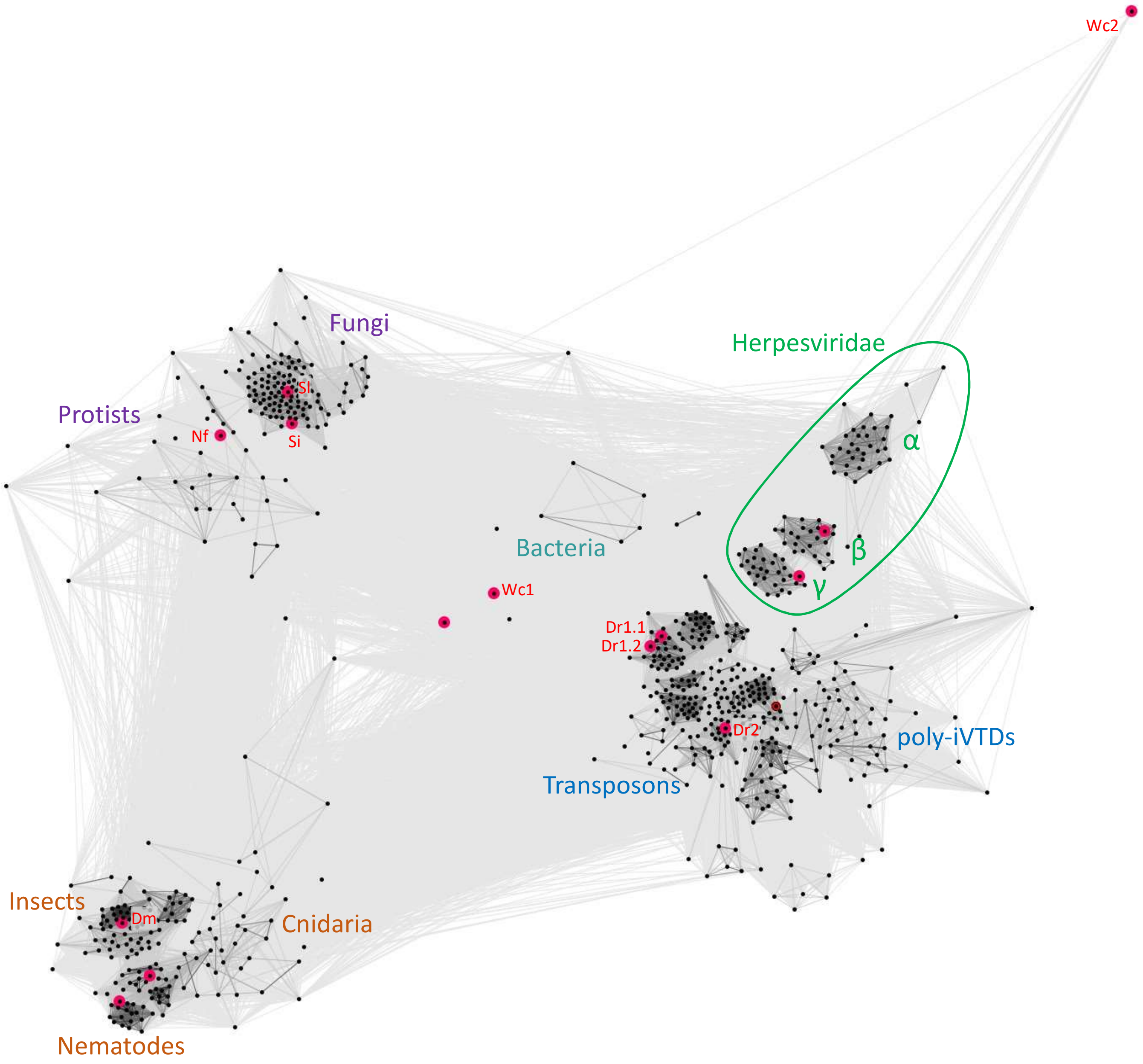


**Supplementary Figure 2**

The sequence relationship between different VTD classes is visualized by similarity-based clustering, using the CLANS software (https://www.eb.tuebingen.mpg.de/protein-evolution/software/clans/). Point-to point distances are approximations of sequence divergence. The herpesviral tegument-DUBs are shown in green (upper-right cluster), the closely related helitron-encoded VTDs and inactive proteins from insects (iVTDs) are shown in blue below the viral cluster. The K6-specific fungal and protist VTDs are shown in purple (upper-left cluster) and the insect and nematode K48-preferring VTDs are shown in brown (lower-left cluster). Wc-VTD1 clusters with other bacterial proteins in the middle portion (colored teal), while the K63-specific Wc-VTD2 is an outlier in the upper right corner.

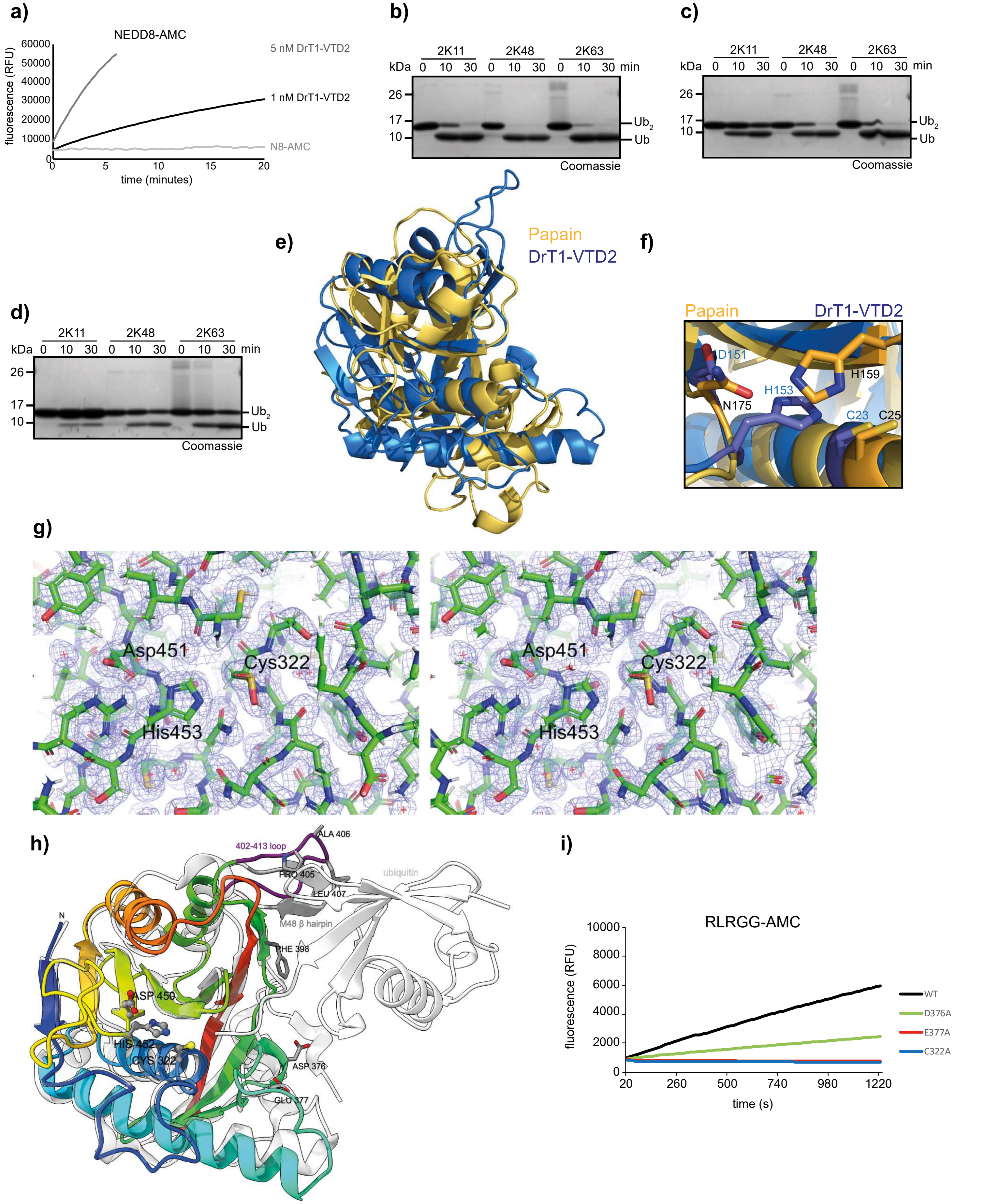


## Supplementary Fig 3

**a)** Activity of 1 nM (black) or 5nM (dark grey) DrT1-VTD2 against NEDD8-AMC. The RFU values are the means of triplicates. **b-d)** Linkage specificity analysis. A panel of K11-, K48- and K63-linked di-ubiquitin chains were incubated with 250 nM DrT1-VTD1 (b), 250 nM DrT1-VTD 2(c) or 25 nM DrT2-VTD (d) for the indicated time points. **e)** Structural superimposition of DrT1-VTD2 (blue) with papain (PDB: 1PPN, yellow). The RMSD is 5.9 Å over 120 residues. **f)** Active site architecture of DrT1-VTD2 (blue sticks) compared to papain (1PPN, yellow sticks). **g)** DRT1-VTD2 active site. The catalytic residues are labelled. The active Cys322 is oxidized to the sulfinic acid. Stereo picture (wall-eye view) of 2Fo-Fc maps (blue colour) contoured at 1 standard deviation above the mean. **h)** Structural superimposition of DrT1-VTD2 (rainbow) and ubiquitin-bound M48 from MCMV (PDB: 2J7Q, grey). **i)** Activity of 5 µM wildtype DrT1-VTD2 (WT, black) against RLRGG-AMC compared to 5 nM of the binding mutants D376A (green), E377A (red) and Δhairpin (blue). The RFU values are the means of triplicates.

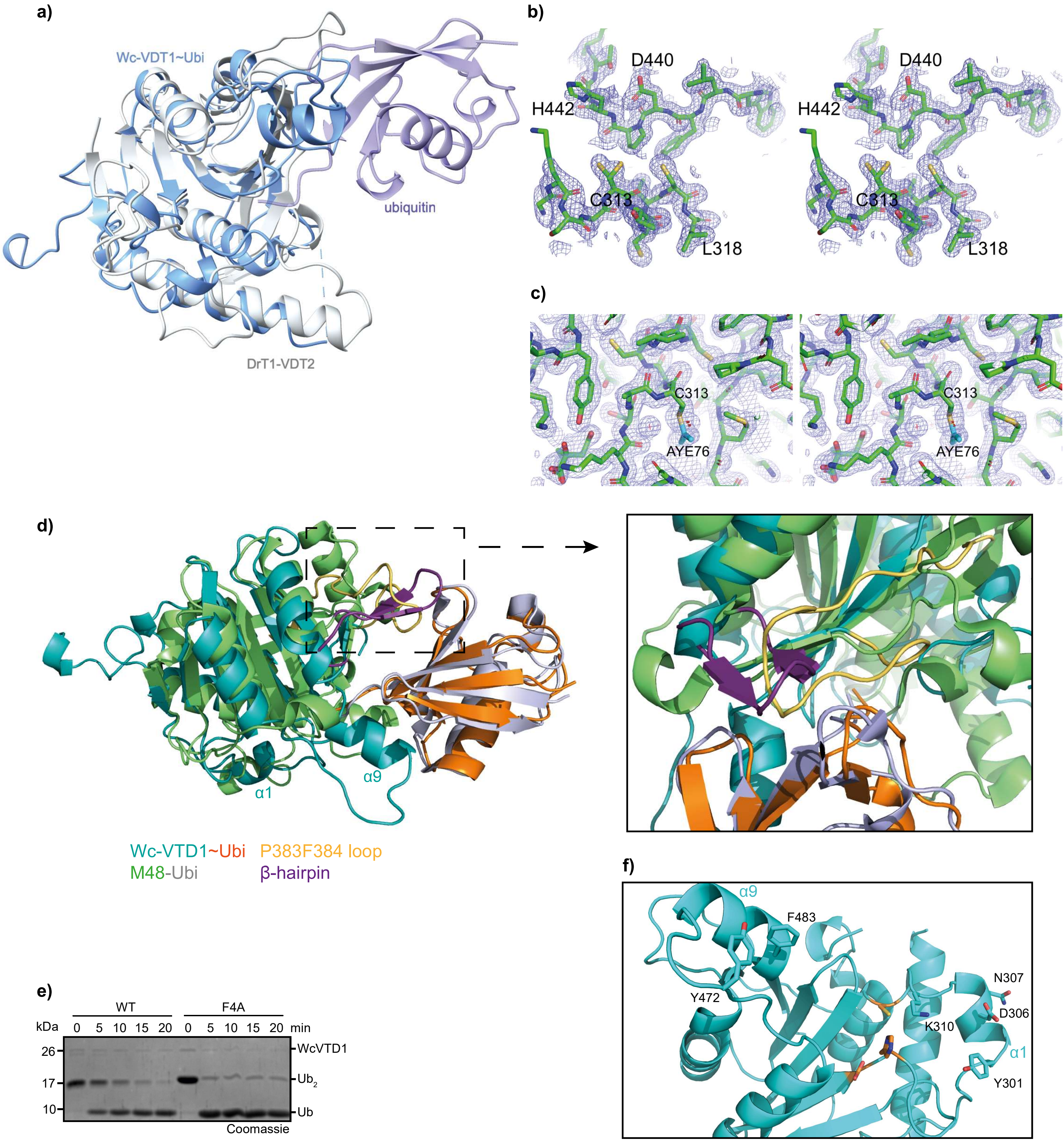


## Supplementary Fig 4

**a)** Structural superimposition of Wc-VTD1-Ub (WC-VTD1 is colored blue, ubiquitin purple) and DrT1-VTD2 (grey). **b, c)** Stereo picture (wall-eye view) of 2Fo-Fc maps (blue colour) contoured at 1 standard deviation above the mean. **b)** Wc-VTD2 in the unbound form. Cys313 has two alternating conformations. The side-chain of His442 is mainly disordered and shown here in an unproductive conformation.**c)** Wc-VTD2 bound to ubiquitin showing the covalent link between Cys313 and the amino-propargyl residue at position 76 of the modified ubiquitin moiety. **d)** The structural superposition of Wc-VTD1-Ub (Wc-VTD1 is colored teal and the ubiquitin orange) and M48-Ub (PDB: 2J7Q, M48 is colored green and the ubiquitin grey) demonstrates different interaction interfaces. The β-hairpin structure of M48 (purple) is absent from Wc-VTD1. The unstructured loop of Wc-VTD1 possibly serving as a replacement is colored yellow. The RMS distance is 1.76 Å over 1113 atoms. **e)** Activity of Wc-VTD1 against wildtype or F4A K6-linked di-ubiquitin. **f)** Surface exposed residues of Wc-VTD1 (colored teal) potentially binding to the proximal ubiquitin are shown as sticks. The active site residues are shown as sticks and colored orange.

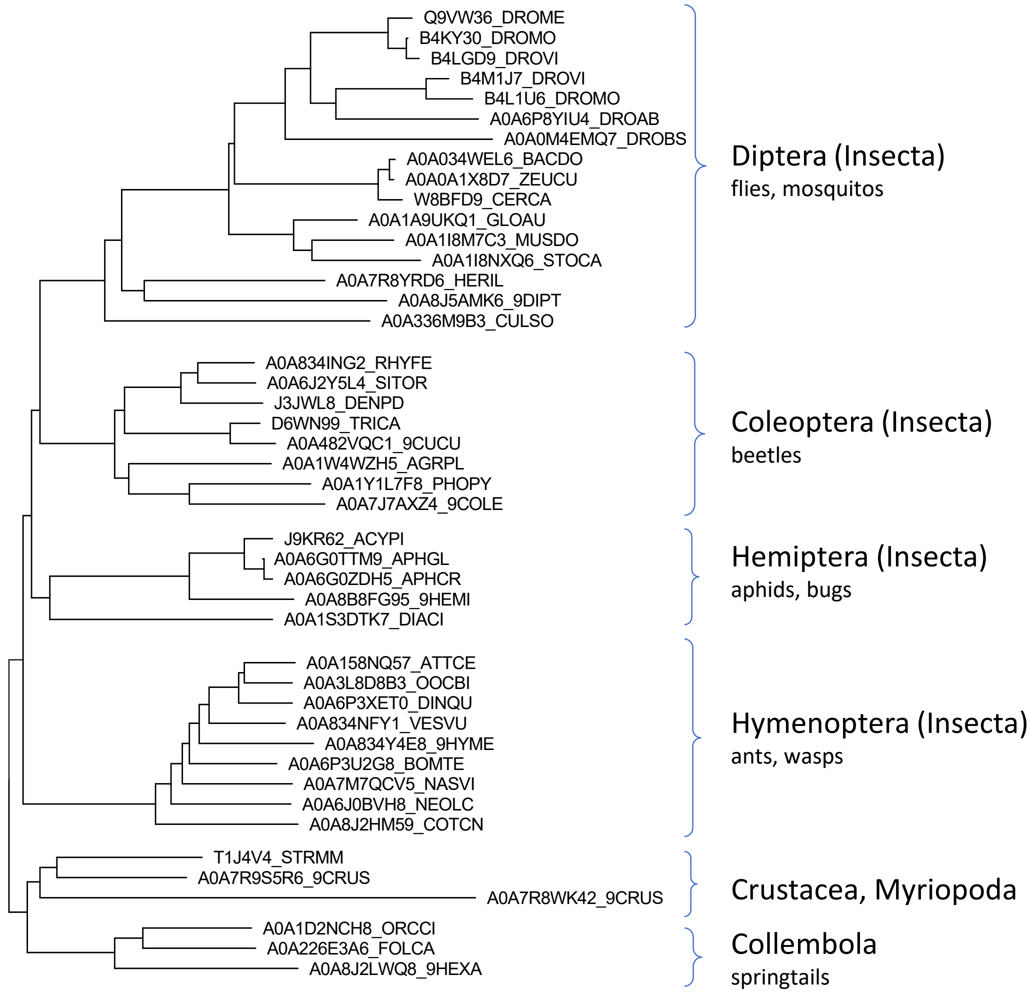


**Supplementary Fig 5**

Neighbor-Joining dendrogram of representative arthropod members of the VTD family. The observed similarity between VTD domains (represented by this dendrogram) roughly recapitulates the phylogenetic ancestry of the host species. Within the depicted insect orders, only some extant species encode VTD proteins. VTDs are completely absent from the order Lepidoptera (moths, butterflies).

a)

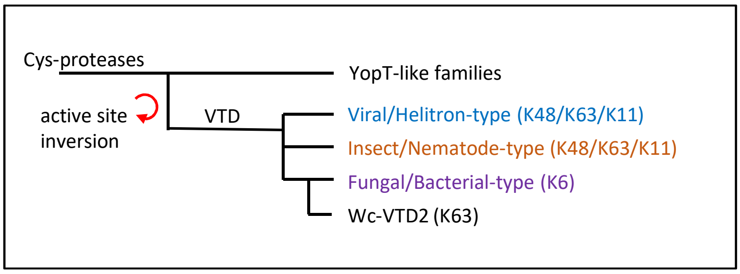


b)

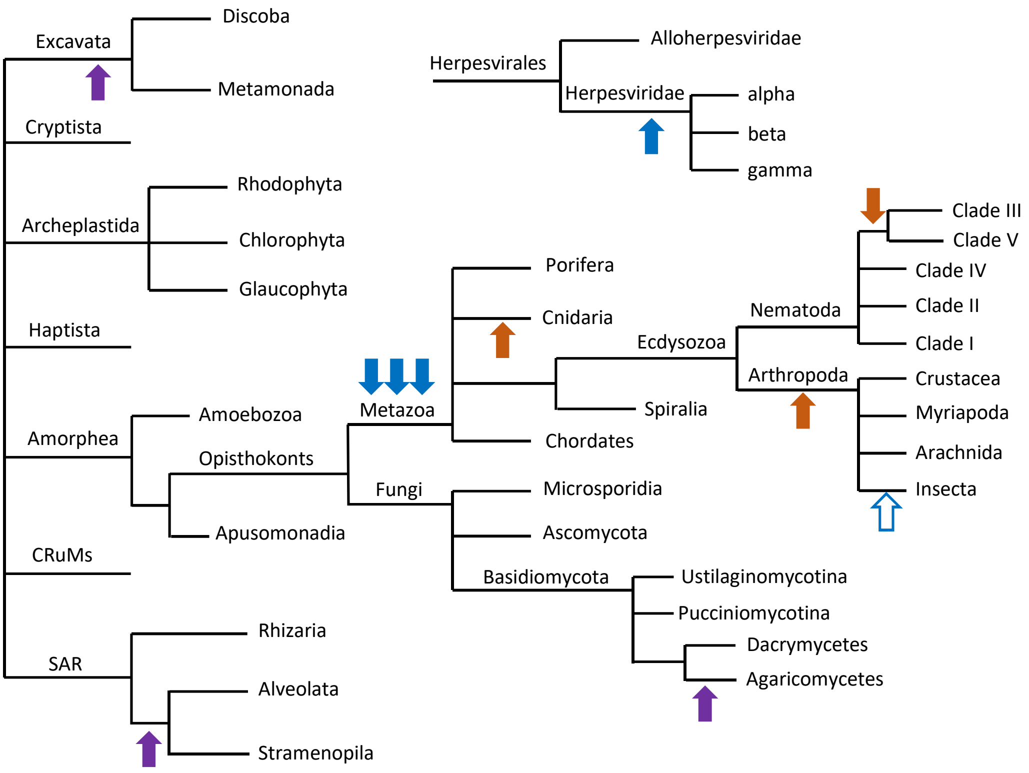


## Supplementary Fig 6

Evolutionary events leading to VTD spread across phyla.

**a)** evolutionary changes with the VTD family, leading to distinct subfamilies (color-coded) with different linkage specificities.

**b)** Simplified eukaryotic tree of life, created using information from https://doi.org/10.1016/j.tree.2019.08.008 and data from the iTOL project https://itol.embl.de (not drawn to scale, only branches relevant to the present analysis are shown. The colored arrows indicate evolutionary events where VTD-proteases were likely acquired. The arrows are color-coded by VTD subfamilies as defined in panel A. The empty arrow indicates integration of an inactive (iVTD) subfamily. The multiple arrows pointing towards the metazoans signify the assumption of multiple helitron-derived VTD integrations into various metazoan lineages.

# Supplementary Information - Raw data

Supplementary Figure 3b

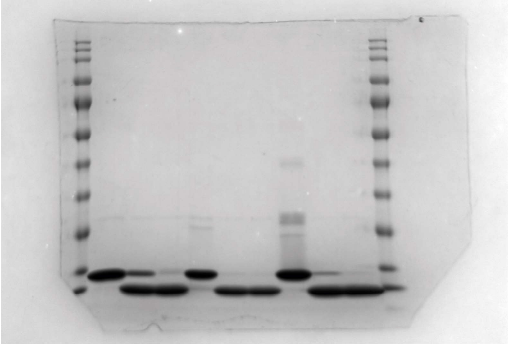

Supplementary Figure 3c

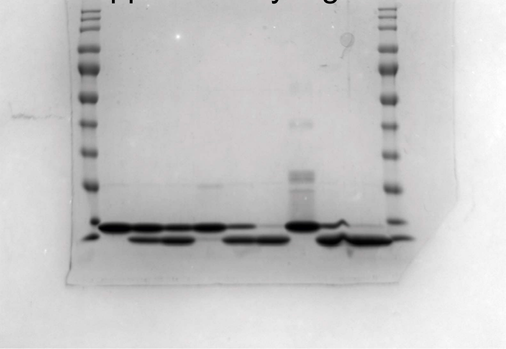

Supplementary Figure 3d

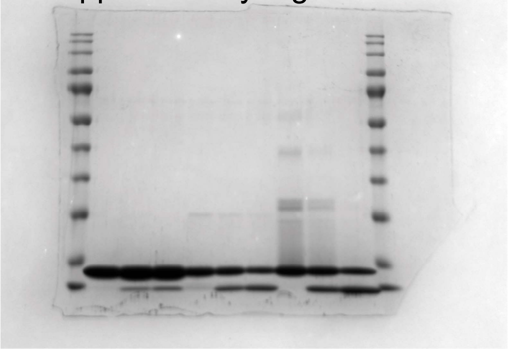

Supplementary Figure 4c

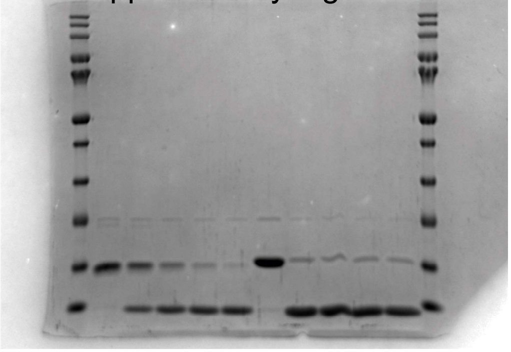

Supplement: Supplementary file 1 — Supplementary Information [file 41467_2022_35244_MOESM1_ESM.pdf]
