# Supplementary material for: A widely distributed family of eukaryotic and bacterial deubiquitinases related to herpesviral large tegument proteins
Source: Nat Commun. 2022 Dec 10;13:7643. doi: 10.1038/s41467-022-35244-y (PMC9741609; doi:10.1038/s41467-022-35244-y)
Supplement: Supplementary file 4 — Source Data [file 41467_2022_35244_MOESM4_ESM.zip › Source_Data_main.pdf]

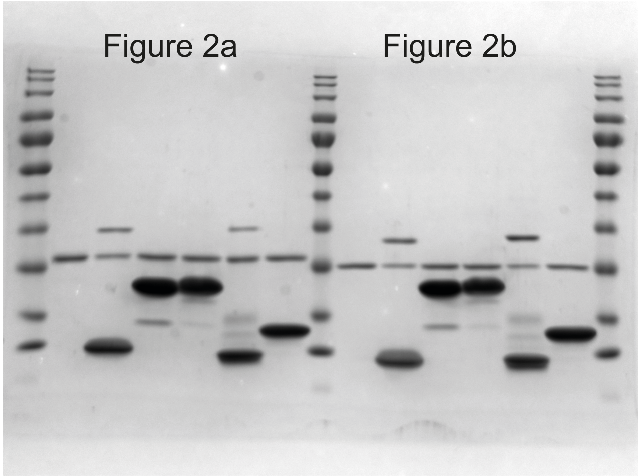

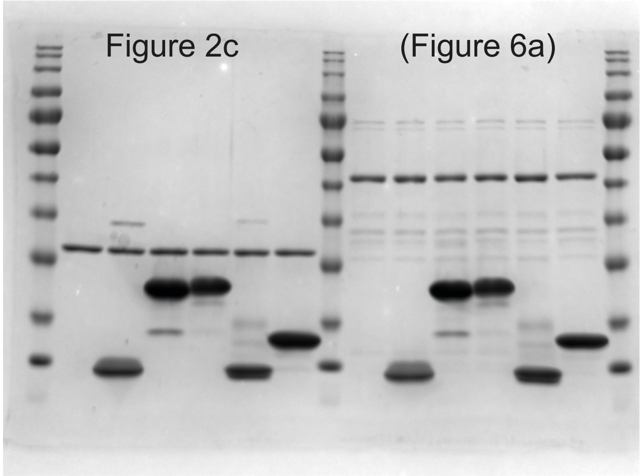

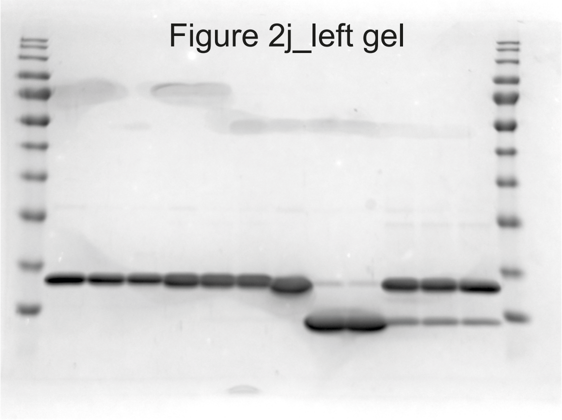

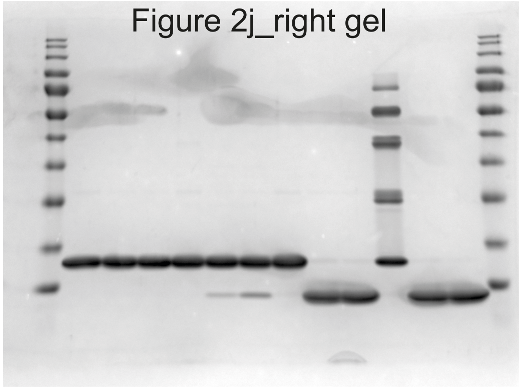

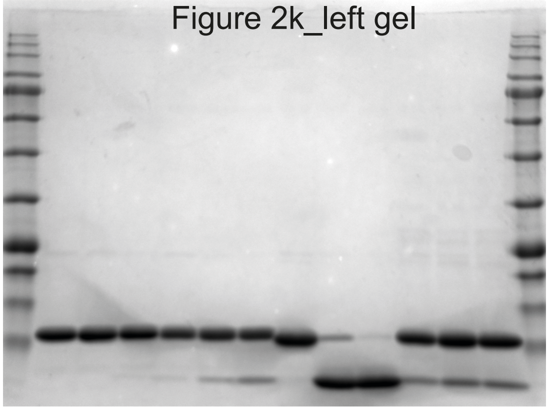

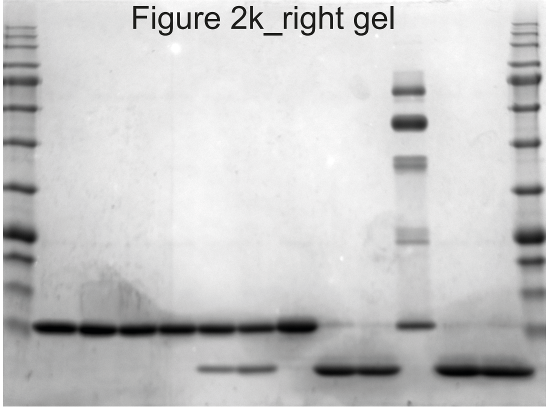

Figure 2l_left gel

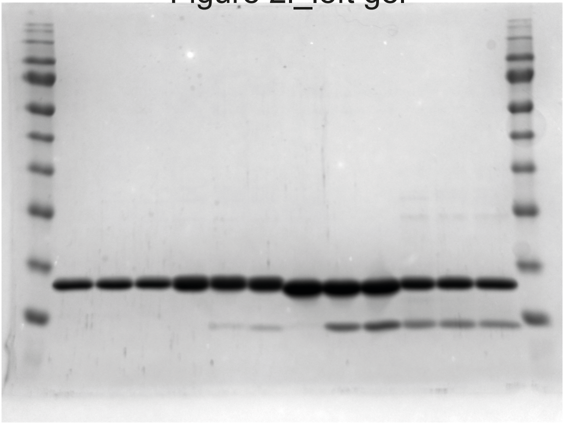

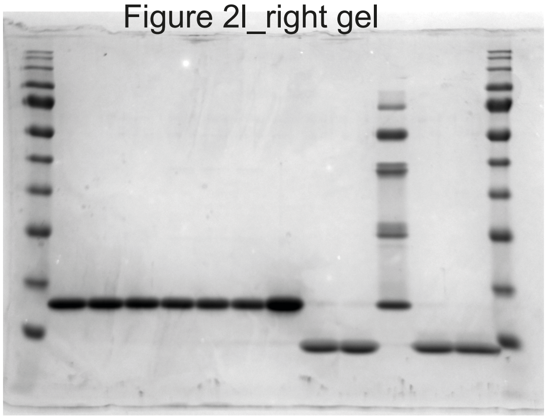

Figure 3f

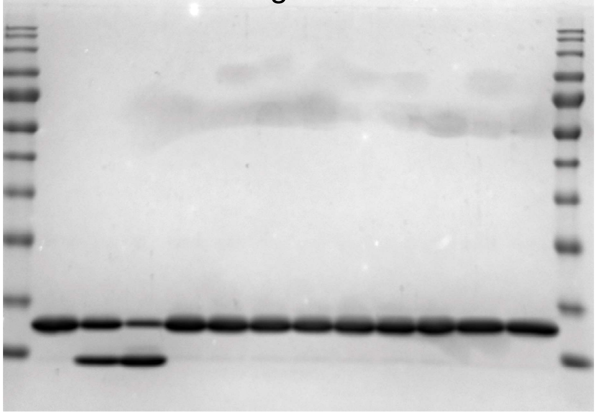

Figure 3i_left gel

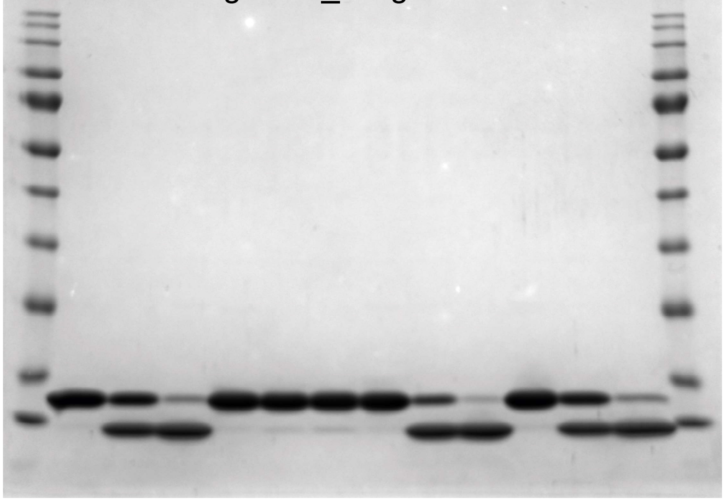

Figure 3i_right gel

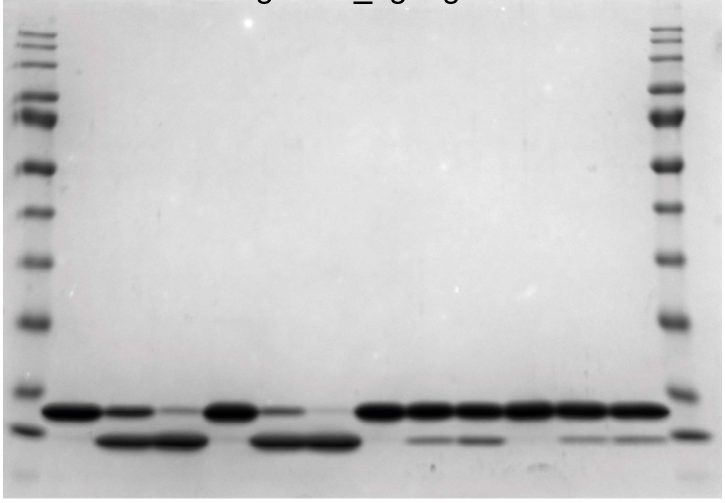

Figure 3j

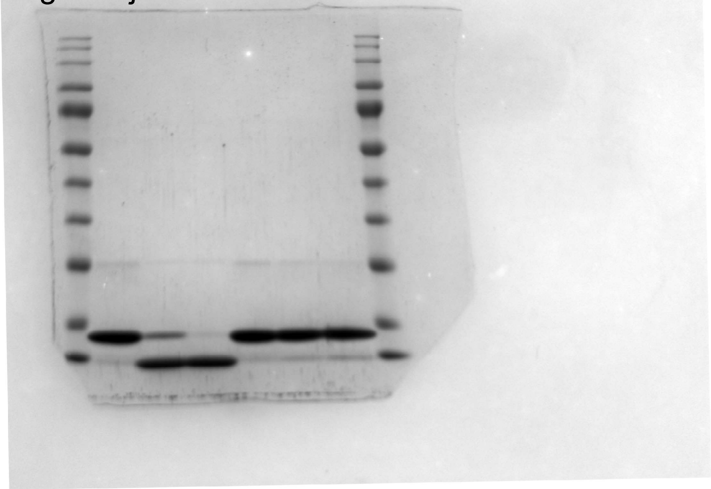

Figure 3k

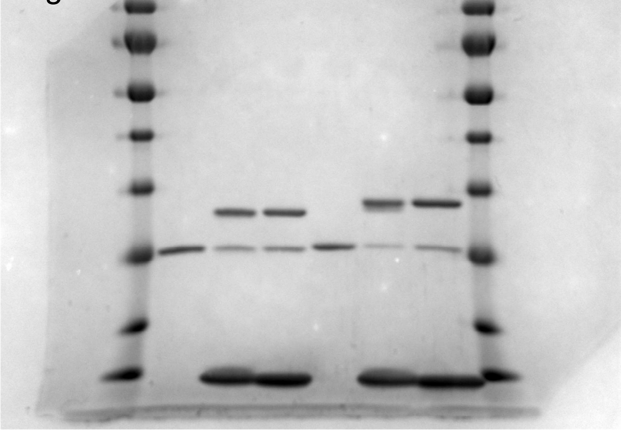

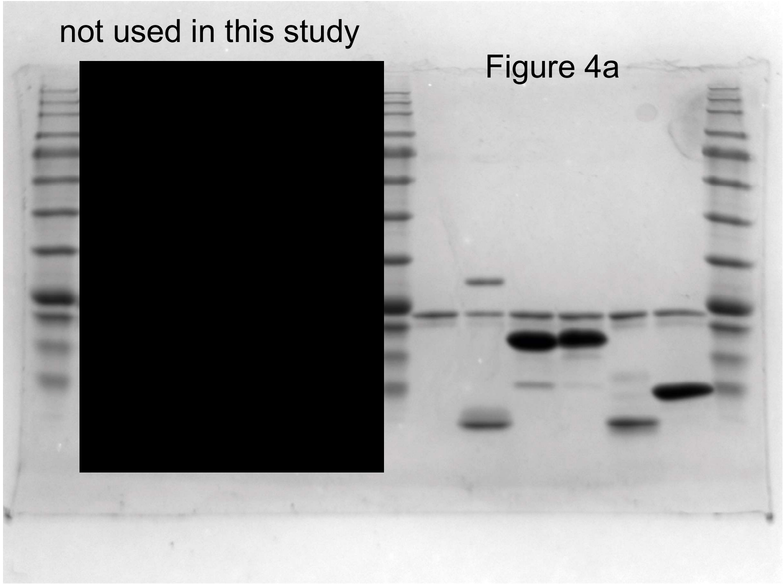
not used in this study
Figure 4a

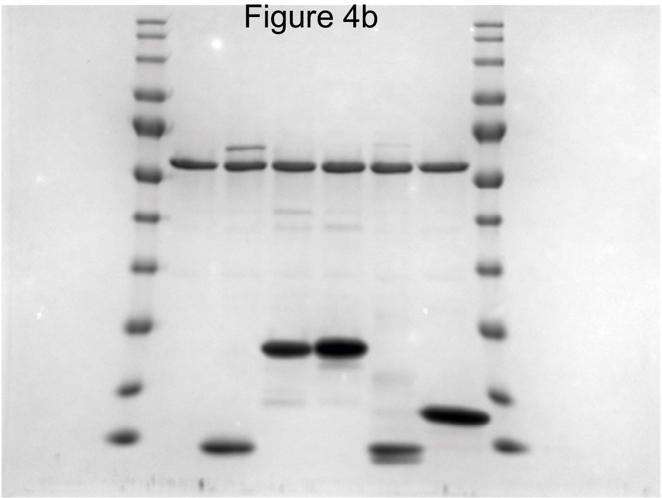
Figure 4b

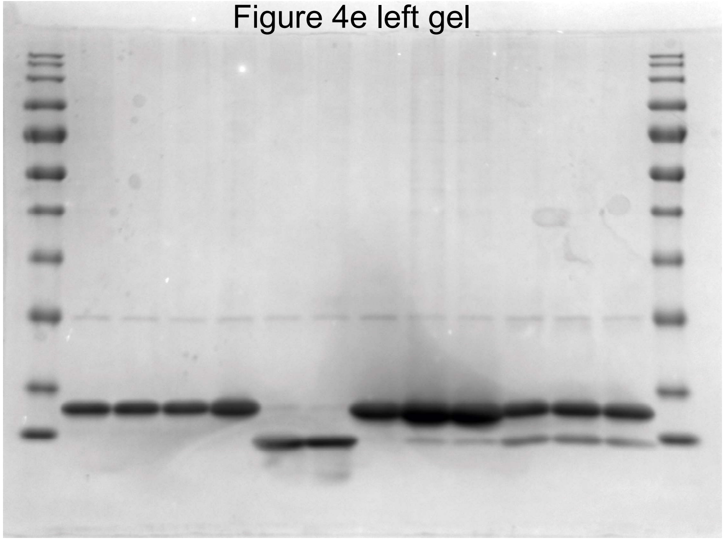
Figure 4e left gel

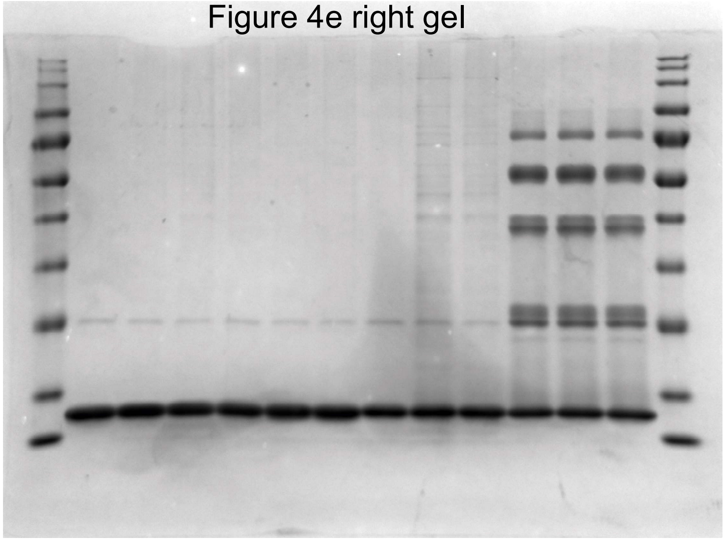
Figure 4e right gel

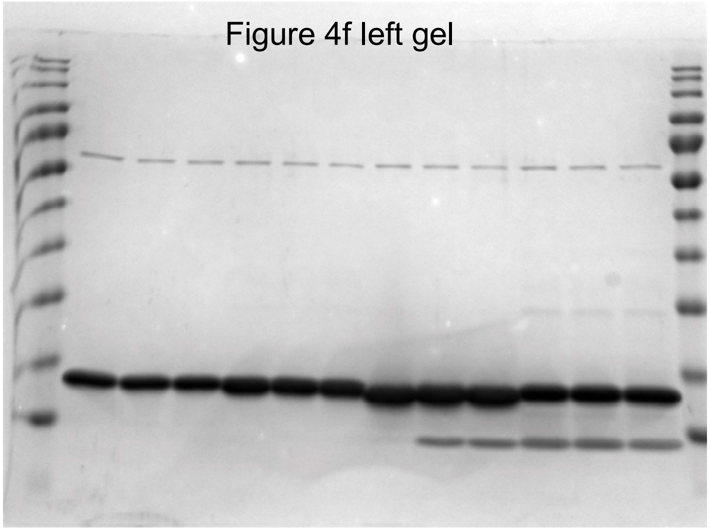
Figure 4f left gel

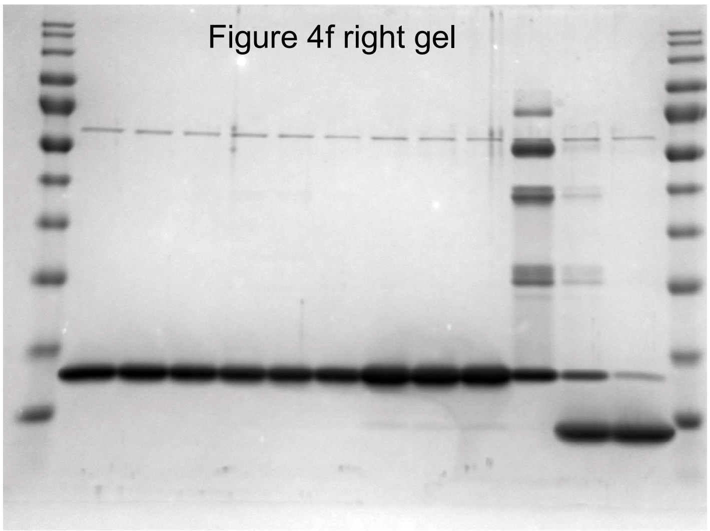
Figure 4f right gel

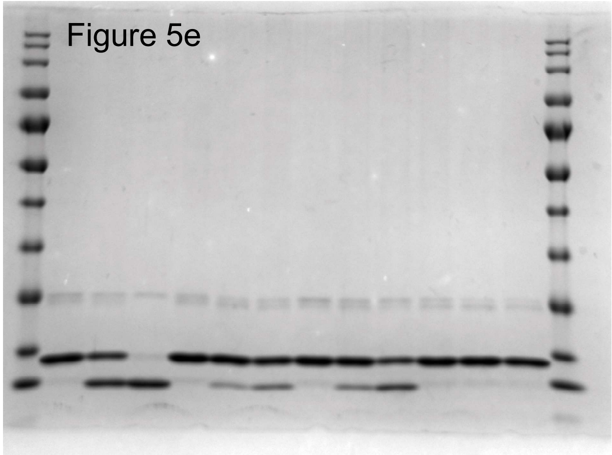

not used in this study

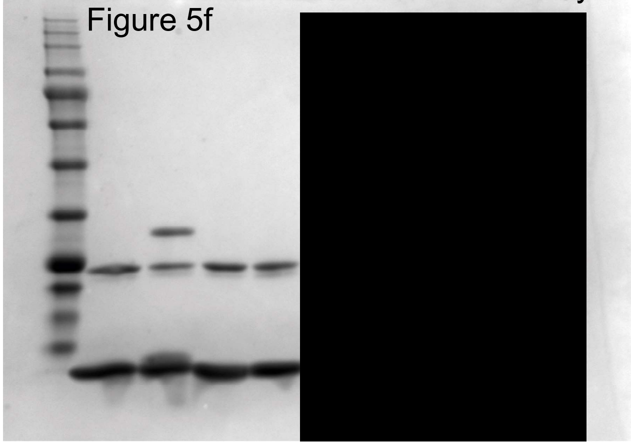

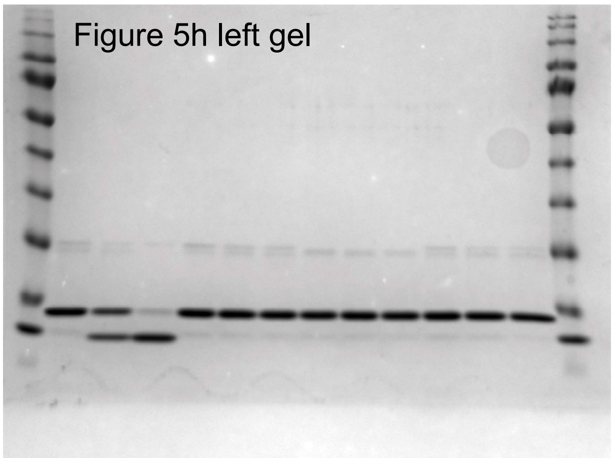

not used in this study

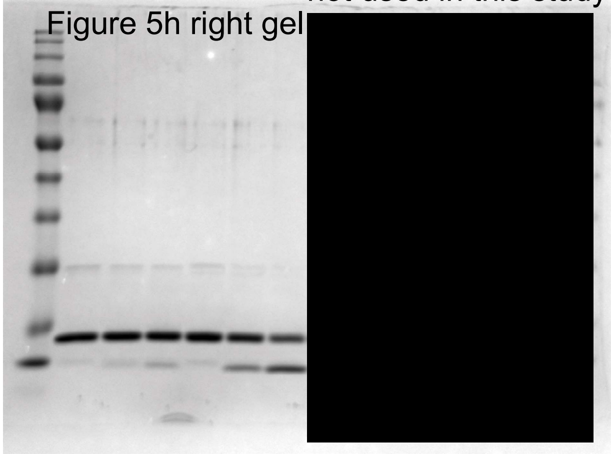

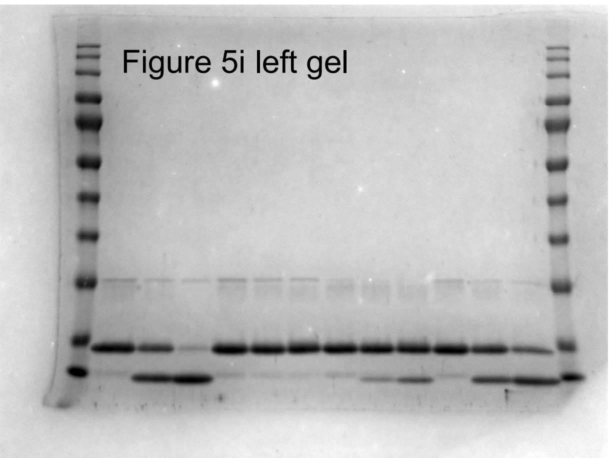

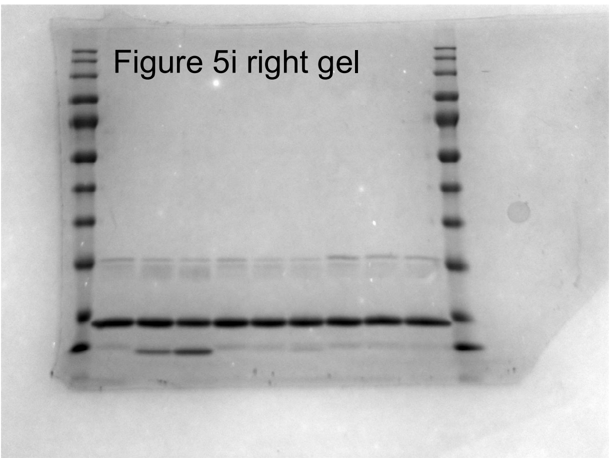

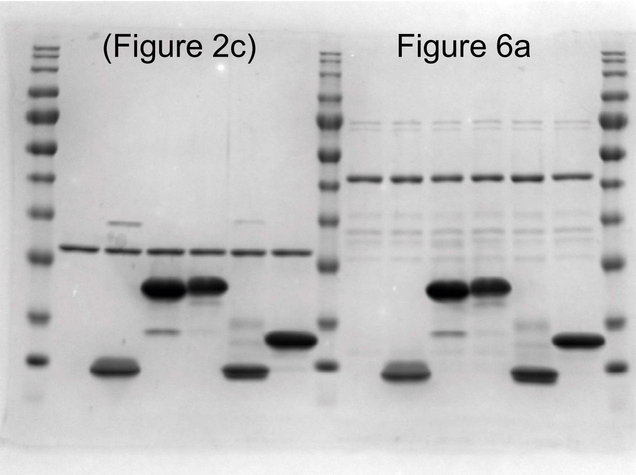
(Figure 2c)
Figure 6a

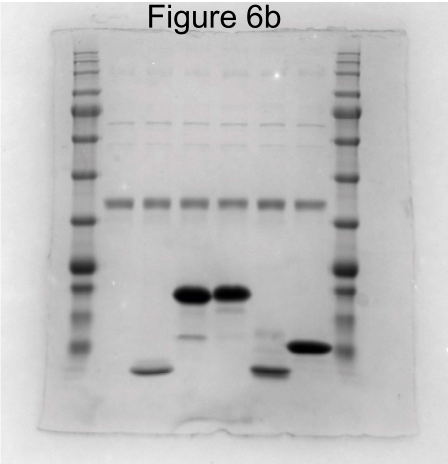
Figure 6b

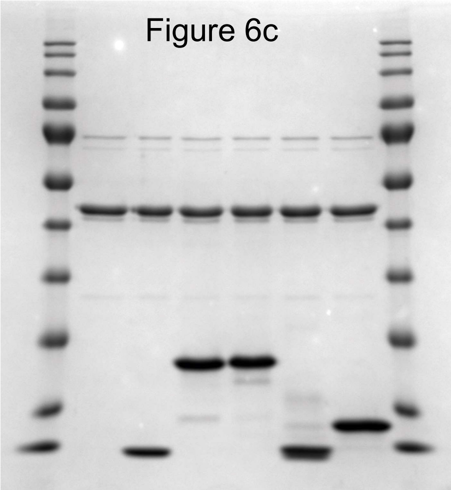
Figure 6c

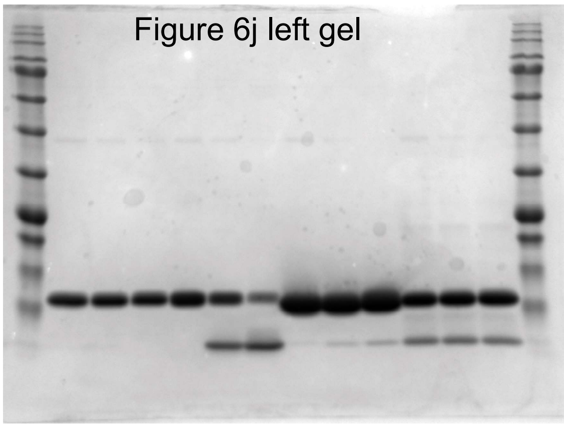
Figure 6j left gel

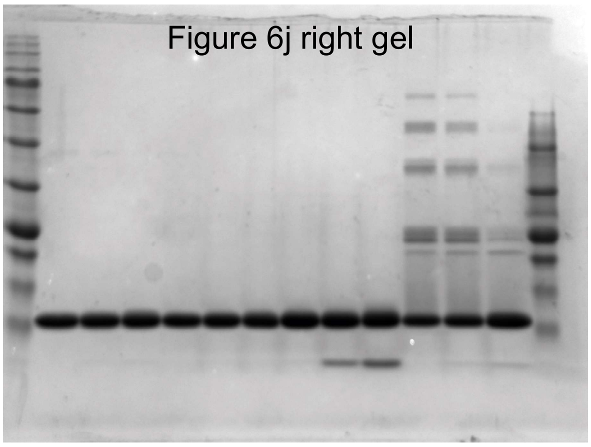
Figure 6j right gel

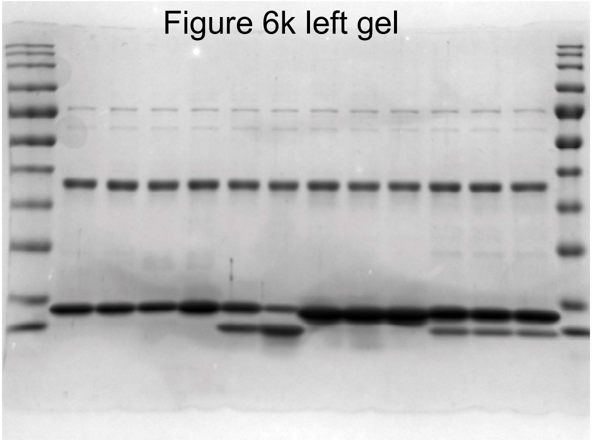
Figure 6k left gel

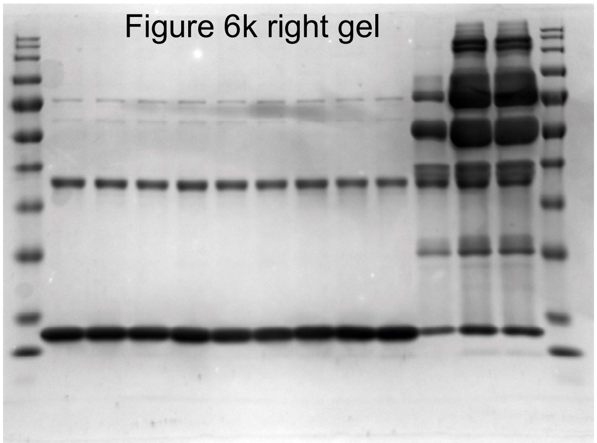
Figure 6k right gel

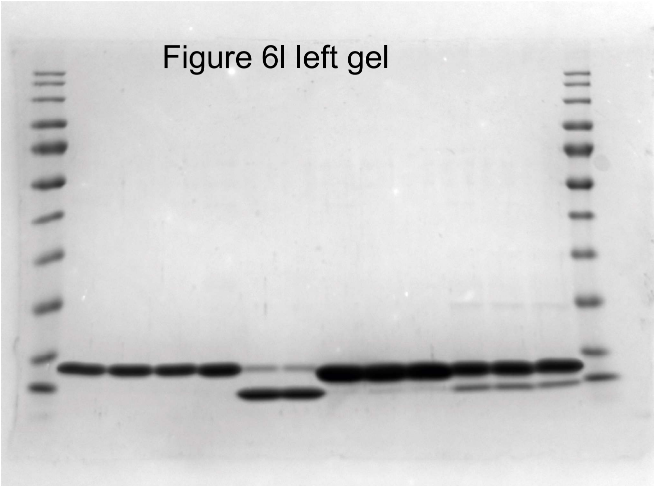
Figure 6l left gel

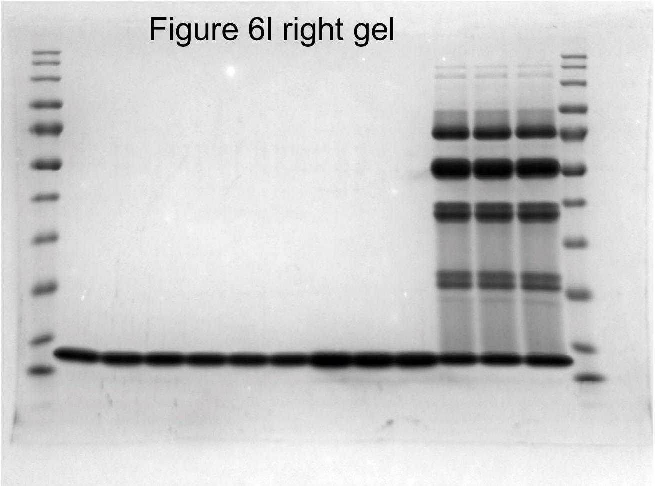
Figure 6l right gel

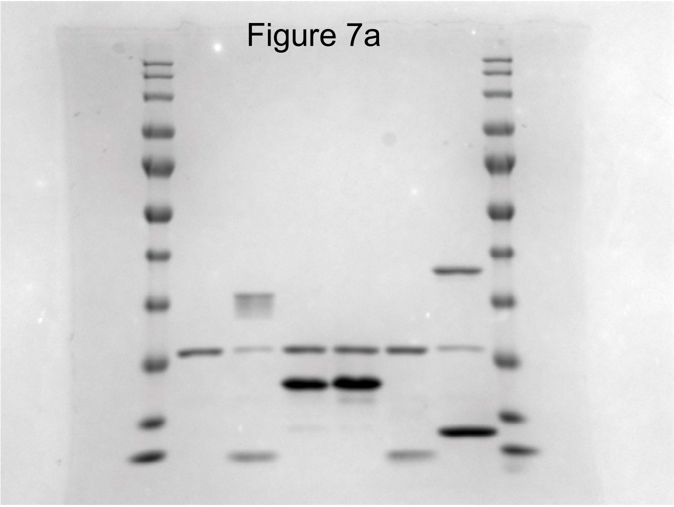
Figure 7a

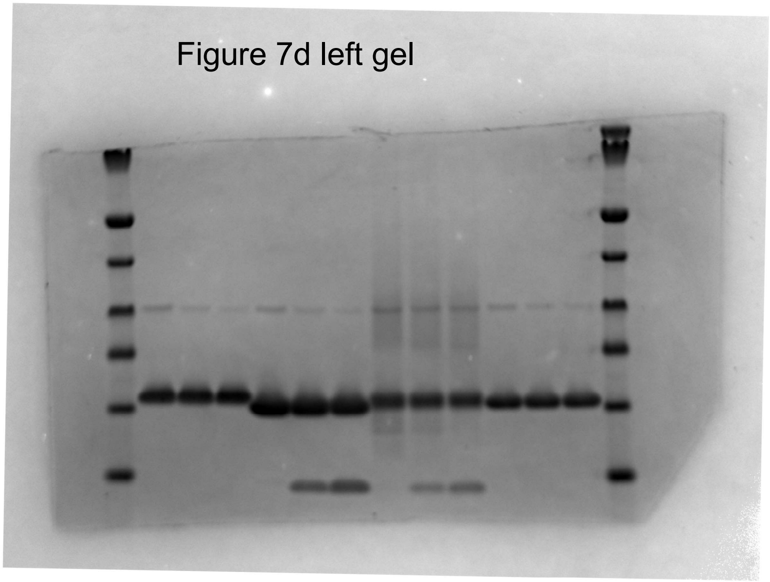
Figure 7d left gel

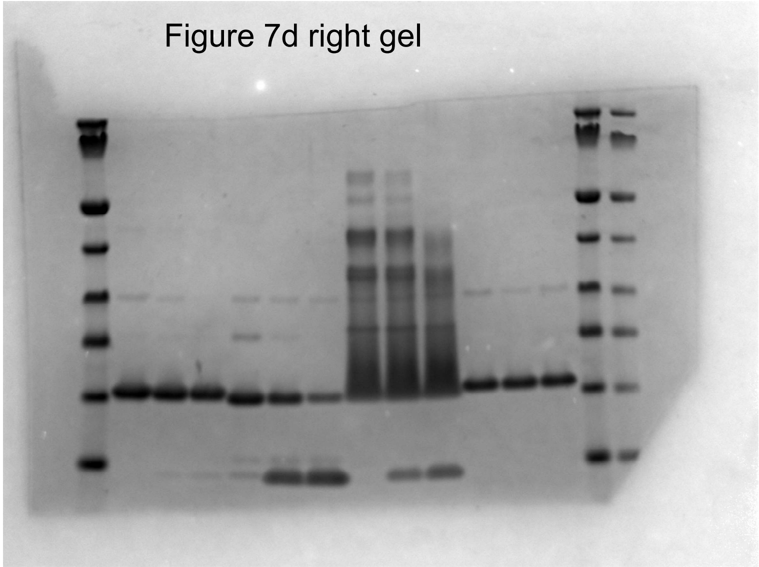
Figure 7d right gel
